# Early nasal and lung transcriptomic profiles reveal pathways associated with divergent clinical outcomes following H7N1 high pathogenicity avian influenza virus infection

**DOI:** 10.1016/j.psj.2026.106833

**Published:** 2026-03-20

**Authors:** María J. Valdez-May, Miquel Nofrarías, Sonia Pina-Pedrero, Marc Dabad, Rosa Valle, Marta Pérez, Anna Esteve-Codina, Jordi Argilaguet, Natàlia Majó, Kateri Bertran

**Affiliations:** a Unitat mixta d’Investigació IRTA-UAB en Sanitat Animal. Centre de Recerca en Sanitat Animal (CReSA). Campus de la Universitat Autònoma de Barcelona (UAB), Bellaterra, 08193, Catalonia, Spain; b IRTA. Programa de Sanitat Animal. Centre de Recerca en Sanitat Animal (CReSA). Campus de la Universitat Autònoma de Barcelona (UAB), Bellaterra, 08193, Catalonia, Spain; c Centre Nacional d'Anàlisi Genòmica (CNAG), Baldiri Reixac 4, 08028, Barcelona, Spain; d Universitat de Barcelona (UB), Barcelona, Spain; e Departament de Sanitat i Anatomia Animals. Facultat de Veterinària. Campus de la Universitat Autònoma de Barcelona (UAB), Bellaterra, 08193, Catalonia, Spain

**Keywords:** Chicken, HPAIV, Resilience, Transcriptomics, MAPK signaling

## Abstract

High pathogenicity avian influenza (HPAI) devastates the poultry industry worldwide due to its rapid spread, severe pathology, and high mortality in chickens. Why some infected birds survive while others die remains poorly understood. Here, we tracked early host transcriptomic responses (8-72 hours post-inoculation [hpi]) in the nasal turbinates and lungs of chickens infected with H7N1 HPAI virus. Chickens were classified as resilient or susceptible based on clinical signs, histopathological lesions, viral antigen detection, and viral shedding. Resilient chickens showed a distinct early transcriptional profile characterized by differential expression of genes related to MAPK signaling (*CAV1*), cell adhesion (*ITGB1, PARVA*), immune response (*RELA*), and antiviral response pathways (*BID, CASP1, RAB2B*). Critically, transcriptomic profiles of resilient birds differed markedly not only from susceptible birds but also from controls, consistent with viral exposure and an active host response. Our results suggest that the nasal mucosa is an important site in which early host responses are associated with divergent disease outcomes following HPAI viral infection.

## Introduction

Avian influenza (**AI**) is a highly impactful viral disease that poses a serious economic challenge to the global poultry industry. In recent years, AI has raised public health concerns due to recurrent outbreaks in mammals and documented human infections (Li et al., 2019; Tiwari et al., 2024). High pathogenicity avian influenza viruses (**HPAIV**) cause rapid systemic dissemination, severe pathology, and high mortality in chickens (Swayne and Suarez, 2000).

The upper respiratory tract serves as the primary entry point for the virus, where the initial host-virus interaction occurs (Leigh Perkins and Swayne, 2002; Pantin-Jackwood and Swayne, 2009; Swayne and Pantin-Jackwood, 2006). Although researchers have studied mammalian respiratory mucosal immunity more extensively, comparable defense mechanisms operate in the avian respiratory tract, including physicochemical and immunological barriers (Esnault et al., 2011; Ohshima, K. and Hiramatsu, K., 2000; Śmiałek et al., 2011). As early as 16 hours following HPAIV exposure, virions actively replicate in the nasal epithelium (Cornelissen et al., 2013; Pantin-Jackwood and Swayne, 2009). Upon viral recognition, epithelial and immune cells initiate antiviral signaling pathways, including the production of interferons, particularly type III interferons, highlighting the relevance of early host responses in the upper respiratory tract after viral infection (Bhowmick et al., 2025; Ranaware et al., 2016; Wells and Coyne, 2018).

HPAIV severity in chickens is influenced by viral and host factors (Matsuu et al. 2016, Pantin-Jackwood and Swayne 2009). Regarding the host, the response to HPAIV can vary among breeds and among individuals within the same species, and transcriptomic studies have identified a molecular signature associated with resilience, mainly in lung tissues (Matsuu et al., 2016; Sánchez-González et al., 2020). Our group previously conducted transcriptomic analysis of lung tissues from HPAIV-resilient and -susceptible chickens and identified genes within the NF-κB pathway as potential early markers associated with the clinical outcome of HPAIV infection. Previous transcriptomic studies of HPAIV resilience have focused on lungs, trachea, or spleen, largely ignoring the nasal turbinates (**NT**), the first site of viral replication. Focusing on the upper respiratory tract at early time points could provide vital insight to halt viral systemic dissemination and inform the development of effective control strategies. In this study, we characterize transcriptomic changes and candidate molecular pathways associated with resilience (becoming infected but overcoming disease) or susceptibility (succumbing to infection) in commercial broiler chickens by analyzing NT and lung tissue samples collected at the early stages of HPAIV experimental infection.

## Materials and methods

### Virus

The HPAIV strain A/Chicken/Italy/5093/1999 (H7N1) was used as challenge virus. This strain was isolated during the HPAI epizootic in Northern Italy in 1999-2000 (kindly provided by Dr. Ana Moreno, *Istituto Zooprofilattico Sperimentale della Lombardia e dell’Emilia Romagna*). This strain's HA0 cleavage site amino acid sequence is PEIPKGSRVRR↓GLF, confirming its high pathogenicity. Virus stocks were propagated and titrated by chorioallantoic sac inoculation of 10-day-old specific pathogen-free (**SPF**) embryonated eggs by standard methods (Woolcock, 2008). This H7N1 HPAIV strain has been routinely used in experimental infections by our group (Bertran et al., 2013; Chaves et al., 2011; Sánchez-González et al., 2020) and, when inoculated at 10^5^ mean egg lethal doses (ELD_50_) via the intranasal route, it causes a consistent mortality rate of approximately 50% of the infected chickens under these experimental conditions, providing an ideal model to compare resilient and susceptible outcomes.

### Animals, housing, and experimental design

Ninety Ross 308 broiler chickens (*Gallus gallus domesticus*) from a commercial producer in Catalonia, Spain, were used. Embryonated chicken eggs were incubated in a commercial hatchery and, once hatched, received a live attenuated Nobilis® Ma5 vaccine against infectious bronchitis virus. Subsequently, 1-day-old chicks were transferred to the *Centre de Recerca en Sanitat Animal* (**IRTA-CReSA**) and housed in a negative-pressure high-efficiency particulate air (**HEPA**) room in the biosafety level 3 (**BSL-3**) facilities with *ad libitum* access to water and feed. After a 7-day acclimation period, birds were individually tagged, and serum, oropharyngeal (**OP**) swabs, and cloacal (**CL**) swabs were obtained from all birds to confirm negativity for AIV by competitive ELISA (ID-VET) and by reverse transcription quantitative PCR (**RT-qPCR**) (Spackman et al., 2002), respectively.

At 15 days old, 10 chickens were used as negative controls and euthanized by cervical dislocation, while the remaining 80 chickens were intranasally inoculated with 10^5^ ELD_50_ of H7N1 HPAIV in 0.05 ml (0.025 ml inoculated in each nostril). All birds were monitored daily for clinical signs throughout the experimental period. Clinical signs were evaluated using a standardized scoring system (WOAH, 2024). Each parameter was rated on a scale from 0 to 3 based on the severity of the clinical signs, including depression, facial edema, diarrhea, respiratory signs, and neurological signs (0: healthy; 1: mild; 2: severe; 3: deceased). For ethical reasons, moribund chickens were euthanized by cervical dislocation and recorded as dead for the following day's scoring. Whole blood in EDTA tubes, OP swabs, and CL swabs were collected from all birds at 8, 24, 48, and 72 hours post-inoculation (**hpi**). Whole blood samples were preserved in TRIzol Reagent (Invitrogen). Swabs were placed in 1 ml of sterile phosphate-buffered saline (PBS) containing 1% penicillin-streptomycin (Thermo Fisher Scientific) and nystatin (Sigma-Aldrich). All samples were stored at -80°C until further use. In addition, 20 inoculated birds were necropsied at 8, 24, 48, and 72 hpi. Tissue samples from the nasal cavity, trachea, lung, brain, heart, spleen, liver, kidney, pancreas, and small intestine were collected in 10% formalin for 72-h fixation. Samples were then embedded in paraffin wax for histopathological evaluation and immunohistochemical (**IHC**) detection of AIV nucleoprotein (**NP**) antigen as previously described (Sánchez-González et al., 2020). NT and lung samples were also preserved in RNAlater (Qiagen) to maintain RNA integrity for subsequent gene expression analysis.

### Reverse transcription quantitative PCR (RT-qPCR) for the detection of HPAIV

RNA from OP and CL swabs was extracted using the IndiMag®Pathogen Kit (INDICAL Bioscience) and the KingFisher Sample Purification System (Thermo Fisher Scientific) following the manufacturer’s instructions. Total RNA was also extracted from whole blood samples using a phenol-based method. The RNeasy Mini kit (Qiagen) was used for purification according to the manufacturer's protocol, with an additional RNase-Free DNase Set (Qiagen) treatment for 15 min at room temperature to eliminate any DNA contamination. In addition, RNA from NT and lung tissue samples collected at 48 hpi was extracted using β-mercaptoethanol and the RNeasy Mini Kit (Qiagen), following the same protocol. Subsequently, a TaqMan RT-qPCR (Heine et al., 2007) was performed using the 7500 Fast Real-Time PCR System (Thermo Fisher Scientific) to quantify viral RNA copies in whole blood samples and in OP and CL swabs. All infected chickens were analyzed, not just those selected for RNA-seq. Statistical analysis was conducted using R (version 4.5.1). Differences in viral detection frequency (Ct < 40) between resilient and susceptible chickens were assessed using Fisher’s exact test. P-values were adjusted for multiple comparisons across tissues within each time point using the Benjamini–Hochberg false discovery rate (**FDR**) correction. Additionally, Ct distribution comparisons were performed as secondary analyses using Wilcoxon rank-sum tests on samples with detectable viral RNA only. In addition, sensitivity analyses were performed in which undetectable samples were assigned the limit of detection (Ct = 40). For NT and lung tissue samples, Ct distributions were compared between resilient and susceptible chickens using Wilcoxon rank-sum tests.

### Classification of chickens as resilient or susceptible

Chickens that fulfilled the following criteria were classified as susceptible: (i) presence of clinical signs (e.g., depression, prostration, and/or dyspnea); (ii) presence of histopathological lesions (including inflammation, hemorrhages, and/or necrosis); (iii) presence of NP antigen in tissues by IHC, associated with histopathological lesions; and (iv) viral detection (Ct < 35) in OP swabs and CL swabs at 48 and 72 hpi. In contrast, birds lacking all four criteria were classified as resilient. Since clinical signs and mortality began at 48 hpi, classification was performed at this time point.

### Host transcriptome analysis (RNA-Seq) on tissue samples

***RNA preparation.*** NT and lung samples (25 mg) collected at 48 and 72 hpi were homogenized in RLT buffer supplemented with β-mercaptoethanol, and total RNA was extracted using the RNeasy Mini Kit (Qiagen), as per the manufacturer’s instructions. Following an RNase-Free DNase Set (Qiagen) treatment for 15 min at room temperature to remove genomic DNA contamination from tissue samples, total RNA was quantified by ultraviolet absorbance at 260 nm using a BioDrop DUO (BioDrop Ltd), and RNA quality was assessed using the RNA 6000 Nano Bioanalyzer 2100 (Agilent). Only samples with RNA integrity number (RIN) > 8 and sufficient RNA quantity were included in downstream transcriptomic analyses. These quality criteria were applied uniformly across all groups. Samples not meeting these thresholds were excluded, resulting in fewer RNA-seq samples than the total number of birds euthanized at each time point. Samples were stored at −80 °C for further processing at the National Center for Genomic Analysis (CNAG, Barcelona, Spain).

***Library preparation and sequencing.*** At CNAG, total RNA was quantified using the Quant-iT Broad-Range dsDNA Assay Kit (Thermo Fisher Scientific), and RNA integrity was assessed using the Fragment Analyzer system HS RNA Kit (15 nt) (Agilent). The preparation of RNA-seq libraries involved the KAPA Stranded mRNA-Seq Kit for Illumina Platforms (Roche), based on the manufacturer’s instructions. Briefly, 500 ng of total RNA was used for poly-A fraction enrichment. The blunt-ended double-stranded cDNA underwent 3′-adenylation, and Illumina platform-compatible adapters with unique dual indexes and unique molecular identifiers (Integrated DNA Technologies) were ligated. The ligation product was then enriched through 15 cycles of PCR, and the final library was validated using an Agilent 2100 Bioanalyzer with the DNA 7500 Assay. Finally, the libraries were sequenced using NovaSeq 6000 system (Illumina) in paired-end mode with a read length of 2 × 51 base pairs, following the manufacturer’s protocol for dual indexing. Image analysis, base calling, and quality scoring of the run were processed using the manufacturer’s Real-Time Analysis (RTA v3.4.4) software, followed by the generation of FASTQ sequence files.

***RNA-seq data processing and analysis.*** Reads were aligned to a combined reference comprising the *Gallus gallus* genome (GRCg7b) and the H7N1 HPAIV genome (GenBank DQ991317–DQ991332) using STAR v2.7.8 (Dobin et al., 2013) with ENCODE-recommended settings for short-read RNA-seq. Gene-level quantification was performed with RSEM v1.3.0 (Li and Dewey, 2011) using default parameters and a reference built from Ensembl release 109 (*Gallus gallus*) plus the H7N1 HPAIV annotation. Differential expression was analyzed with limma v3.42.3 (Ritchie et al., 2015), counts were TMM-normalized, transformed with voom (Law et al., 2014) to log2 counts-per-million with precision weights, and modeled with linear models including group (HPAIV-susceptible, HPAIV-resilient, control) and sex as fixed effects. Empirical Bayes moderation was applied, and *P*-values were adjusted by Benjamini–Hochberg. Genes with FDR < 0.05 in comparisons between each experimental group and the control group were considered differentially expressed. Principal component analysis (**PCA**) was performed on the voom-transformed log2 counts-per-million matrix to assess sample structure and screen for potential outliers. PCA was computed on the top 500 most variable genes, and components were examined for clustering by phenotype (HPAIV-resilient, HPAIV-susceptible, control). Functional over-representation analysis of differentially expressed genes (**DEGs**) was carried out with DAVID v6.8 (http://david.ncifcrf.gov/; accessed 1 August 2024) (Huang et al., 2009). KEGG pathway enrichment (Kanehisa et al., 2021) was queried via DAVID using the set of DEGs from each contrast and a background defined as all genes tested in the differential expression analysis. *P*-values were adjusted for multiple testing using the Benjamini–Hochberg procedure. All plots were generated in R v4.5.1 using ggplot2 (Wickham, 2016) and pheatmap (Kolde and Kolde, 2015). Gene expression values were scaled by row (z-score transformation) prior to visualization. Hierarchical clustering was performed on both rows (genes) and columns (samples) using Euclidean distance and complete linkage.

Spearman’s rank correlation analysis was applied to assess the association between the expression levels of selected candidate genes and viral load in NT and lung samples at 48 hpi. RT-qPCR Ct values were used as a relative proxy for viral RNA levels. Correlation analyses were performed in R v4.5.1.

### Microfluidic quantitative PCR assay (PCR Multiplex Fluidigm)

A list of unique candidate genes associated with resilience was generated by integrating DEGs and pathways identified in NT and lungs of resilient chickens at 48 and 72 hpi. We prioritized genes involved in immune regulation and antiviral response pathways, as well as additional DEGs displaying biologically relevant expression changes (high positive or negative logFC) that were not categorized by DAVID but could participate in host–virus interactions. Furthermore, we included interferon-stimulated genes (**ISGs**) previously associated with influenza A virus infection. Primer design and validation were carried out according to established criteria (Te et al., 2021). A 48 x 48 Dynamic Array IFC was used to analyze the expression of these genes. The total RNA was reverse-transcribed to cDNA using the PrimeScript RT reagent kit (Takara Bio, Inc.) according to the manufacturer’s instructions. Briefly, cDNA samples were preamplified using a Preamp Master Mix (Fluidigm Corporation) for 16 cycles. Subsequently, a cleanup was performed with Exonuclease I (New England Biolabs), Reaction Buffer (New England Biolabs), and nuclease-free water. The Biomark HD system (Fluidigm Corporation) was used to obtain gene expression levels. Data analysis was performed using the Standard Biotools Real-Time PCR analysis software v1.02 (Fluidigm Corporation) and the DAG expression software v1.0.5.6 (Ballester et al., 2013). Finally, gene expression levels were normalized using the reference genes *RPL13, RPL27A*, and *RPL17*.

Virological parameters (viremia and viral shedding) were first assessed at 24 hpi in all infected birds and then compared between birds later classified as resilient or susceptible according to clinical and pathological criteria. Based on these results, the expression of selected genes (Supplementary Table 1) was analyzed in NT and lung samples collected from birds euthanized at 24 hpi (before the onset of clinical signs) to evaluate whether early transcriptomic differences preceded divergent disease outcomes. At 24 hpi, NT and lung samples from resilient (n = 5) and susceptible (n = 5) birds were analyzed. Moreover, the same selected genes were evaluated in NT samples collected at 48 hpi, including a subset of samples used for RNA-seq, to corroborate the transcriptomic findings (Supplementary Table 1). At 48 hpi, NT samples from resilient (n = 6) and susceptible (n = 4) birds were analyzed.

***Statistical analysis.*** All statistical analyses were performed using R v4.5.1, and heatmaps were generated using the pheatmap package (Kolde & Kolde, 2015). Welch’s one-way ANOVA was used to assess statistical differences among groups. To account for multiple testing across the genes analyzed, the resulting p-values were adjusted using the Benjamini–Hochberg FDR correction. Genes with an adjusted p-value (FDR) ≤ 0.05 were considered statistically significant.

## Results

### Significant differences in viremia and viral shedding between resilient and susceptible chickens are evident before the onset of clinical signs and mortality

**Fig. 1:**
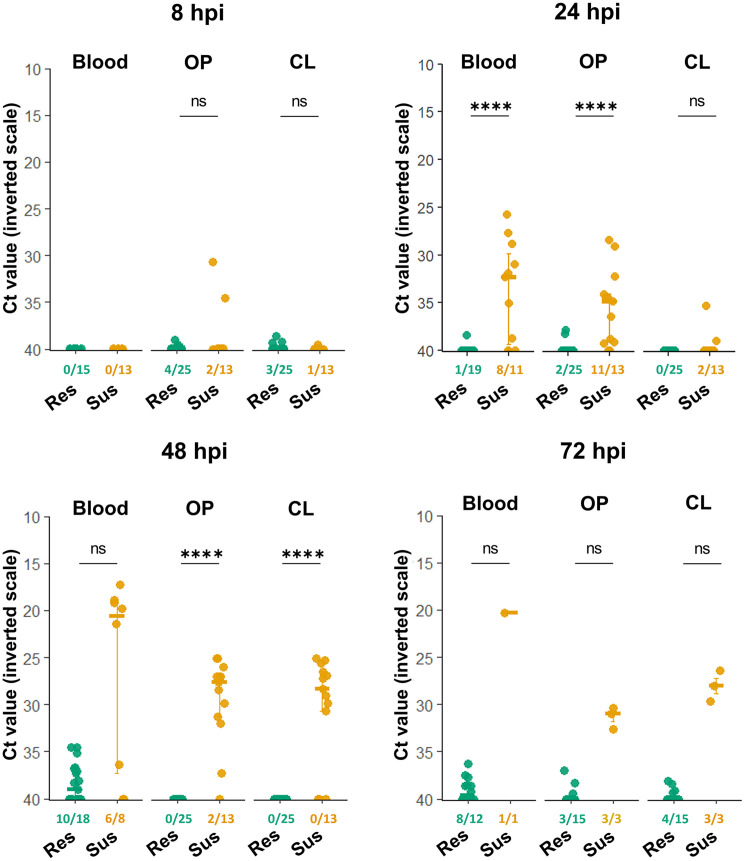
RT-q**PCR of the M gene from whole blood, oropharyngeal (OP) swabs, and cloacal (CL) swabs at 8, 24, 48, and 72 hours post-inoculation (hpi), obtained from resilient (Res) and susceptible (Sus) H7N1 HPAIV inoculated chickens**. Each dot represents an individual bird; horizontal lines indicate the median, and error bars represent the interquartile range (IQR). The limit of detection (LOD) was defined as Ct = 40. Samples with Ct < 40 were considered positive for detectable viral RNA. For ease of interpretation, the Ct axis is inverted so that lower Ct values, corresponding to higher viral RNA levels, appear higher on the graph. The ratios indicate the number of samples with detectable viral RNA relative to the total number of samples. Differences in detection frequency (Ct < 40) between groups were assessed using Fisher’s exact test. P-values were adjusted for multiple comparisons across tissues within each time point using the Benjamini–Hochberg procedure. ns (not significant), *p ≤ 0.05, **p ≤ 0.01, ***p ≤ 0.001, and ****p ≤ 0.0001 (BH-adjusted p-values). Comparisons of Ct distributions were performed as secondary analyses using Wilcoxon rank-sum tests on detectable samples only and as sensitivity analyses using LOD-imputed values (Ct = 40).

Importantly, in this study, the term “resilient” refers to birds with a favorable clinical-pathological course, as defined by the criteria above. Therefore, this designation does not imply inherent resistance to HPAIV infection. In line with this definition, resilient and susceptible chickens shed virus at markedly different rates (Fig. 1). Susceptible chickens shed virus as early as 8 hpi, predominantly through the OP route. The peak of viral shedding occurred at 48 hpi, as evidenced by both the number of birds shedding and the viral copies detected in OP and CL swabs. By 72 hpi, all surviving susceptible chickens were shedding the virus through both OP and CL routes. In contrast, resilient chickens shed low levels of viral RNA at all time points. Similarly, viremia levels were higher in susceptible birds than in resilient birds. At 24 hpi, viral RNA was detected in the blood of susceptible chickens, whereas in resilient chickens, it was undetectable or detected at very low levels (Fig. 1). Notably, susceptible birds showed higher viral RNA levels than resilient birds as early as 24 hpi in both blood and OP samples, preceding the onset of clinical signs and mortality, which occurred around 48 hpi. These results suggest early virological measurements might help predict disease outcomes.

### Transcriptomic profiles reflect predefined clinical outcome groups

Based on predefined clinical and pathological criteria, a total of 14 chickens were classified as HPAIV-susceptible and 25 as HPAIV-resilient. One bird was excluded due to unscheduled euthanasia, resulting in a total of 39 chickens. At 48 hpi, the 20 euthanized chickens included 10 susceptible and 10 resilient birds. By 72 hpi, four birds were classified as susceptible and 16 as resilient.

**Fig. 2:**
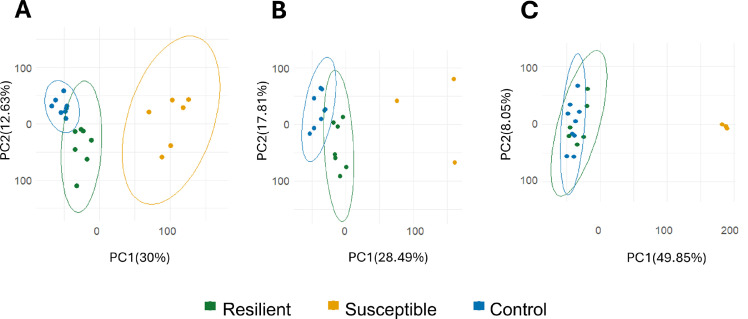
**Principal component analysis (PCA) of gene expression data from NT at 48 hours post-inoculation (hpi) (A), NT at 72 hpi (B), and lungs at 48 hpi (C) from HPAIV-resilient, -susceptible, and control (0 hpi) chickens.** The analysis was performed on log-transformed data, normalized using the limma-voom method. Each point represents a sample, color-coded by experimental group (green for resilient, orange for susceptible, and blue for control). The ellipses represent the 95% confidence intervals for each group. PC1 and PC2 represent the first and second principal components, respectively, explaining the percentage of variance in the dataset (represented with percentages).

The final dataset of transcriptomic analysis at 48 hpi included: i) NT from eight controls, seven resilient, and six susceptible birds; and ii) lungs from nine controls, five resilient, and three susceptible birds. At 72 hpi, NT from seven resilient and three susceptible birds were analyzed, but lung samples were not included in the analysis (Supplementary Table 2). PCA showed that transcriptomic profiles were consistent with the clinical outcome groups defined by clinical and pathological criteria (Fig. 2). However, while the NT profiles of resilient birds could still be distinguished from controls (Fig. 2**A, B**), the lung profiles of resilient and control birds were not clearly separated (Fig. 2**C**). The clustering of transcriptomic profiles by clinical outcome suggests that our classification criteria captured biologically meaningful differences.

### Viral burden and immune transcriptomic profiles distinguish HPAIV-resilient from -susceptible chickens

**Fig. 3:**
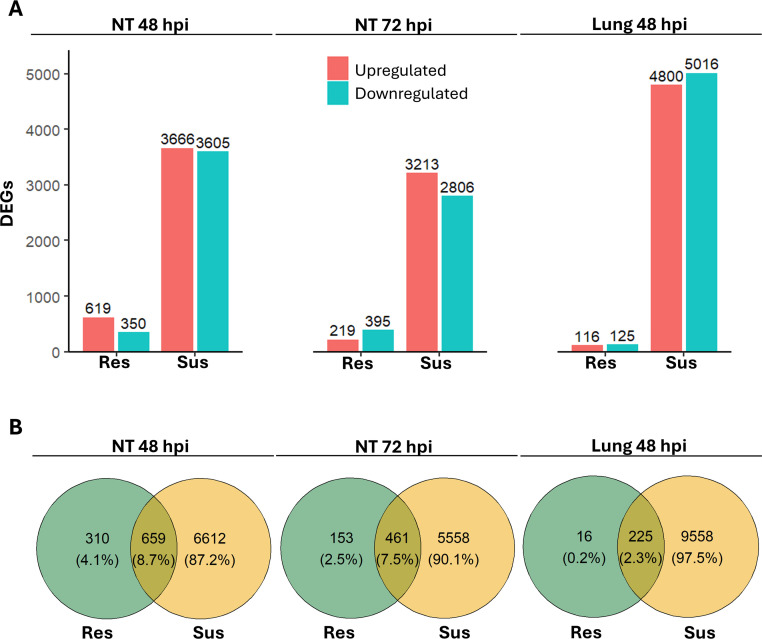
**Transcriptomic analysis of NT and lung samples from HPAIV-resilient and -susceptible chickens at 48 and 72 hours post-inoculation (hpi). (A)** Number of upregulated and downregulated DEGs in HPAIV-resilient and -susceptible groups compared with controls. **(B)** Venn diagram illustrating the overlap and unique DEGs between HPAIV-resilient and HPAIV-susceptible groups.

Transcriptomic analysis of NT and lung samples from resilient and susceptible chickens was conducted to characterize the host determinants associated with each clinical outcome. Although the two groups of chickens showed DEGs compared to controls in both tissues, susceptible birds showed more DEGs than resilient birds. This finding is consistent with the higher viral RNA counts detected in susceptible chickens (Supplementary Fig. 1A). Moreover, although viral shedding was low or undetectable by molecular methods in swabs from resilient birds, viral RNA was nonetheless detected in both NT and lung tissues at 48 hpi using RNA-seq and RT-qPCR. This is consistent with resilient birds having been exposed to the virus and with viral RNA being present in the respiratory tract, with RNA levels slightly higher in NT than in lung samples (Supplementary Fig. 1A, B). The number of upregulated and downregulated DEGs was similar across samples (Fig. 3**A**). Notably, only 0–4% of DEGs were unique to resilient chickens (Fig. 3**B**), indicating that most transcriptional changes were shared between the two clinical outcomes (Fig. 3**B**). However, these genes showed moderate-to-strong effect sizes, especially in NT at 48 hpi.

Functional enrichment analysis was conducted for both resilient and susceptible groups. KEGG pathways such as “Influenza A” (gga05164), “Toll-like receptor signaling pathway” (gga04620), “RIG-I-like receptor signaling pathway” (gga04622), and “NOD-like receptor signaling pathway” (gga04621) were identified in upregulated DEGs from both NT and lung samples in susceptible chickens at 48 and 72 hpi (Supplementary Fig. 2A). By contrast, the downregulated DEGs in both tissues of susceptible birds were primarily associated with metabolic pathways (gga01100) (Supplementary Fig. 2B). “cell migration” (GO:0016477), “cell-cell adhesion” (GO:0098609), and “Focal adhesion” (gga04510) were identified in downregulated DEGs in lung samples from susceptible chickens (Supplementary Fig. 2B).

In the NT of resilient chickens, functional enrichment analysis of upregulated genes identified biological processes such as “Endocytosis” (gga04144), “positive regulation of cell migration” (GO:0030335), “Focal adhesion” (gga04510), and “negative regulation of NF-κB signal transduction” (GO:0019221) at 48 hpi, as well as “negative regulation of MAPK cascade” (GO:0043409) at 72 hpi. In lung samples, upregulated DEGs at 48 hpi were associated with “positive regulation of receptor-mediated endocytosis” (GO:0048260) and “intracellular iron ion homeostasis” (GO:0006879) (Supplementary Fig. 3A).

Conversely, the analysis of all downregulated genes identified terms such as “Metabolic pathways” (gga01100) and “protein transport” (GO:0015031) in NT at both time points, along with “regulated exocytosis” (GO:0045055) only at 48 hpi in NT. Moreover, lungs at 48 hpi exhibited terms such as “inflammatory response to antigenic stimulus” (GO:0002437) and “negative regulation of stress-activated MAPK cascade” (GO:0032873) (Supplementary Fig. 3B).

To characterize the immunological mechanisms differentiating resilient from susceptible birds, we examined genes involved in interferon signaling, apoptosis, and inflammation. The gene expression patterns in NT and lung samples at 48 and 72 hpi showed a clear contrast between resilient and susceptible birds in key pathways associated with HPAIV response (Supplementary Fig. 4). Moreover, susceptible chickens displayed an upregulation of immune-related genes compared with controls, including genes involved in apoptosis (e.g., *MAPK14, CASP7, CASP3*), interferon response (e.g., *IRF7, MX1*), and inflammation (e.g., *IL6, IL8L2, TRIM25*) in both the NT and lung at 48 and 72 hpi (Supplementary Fig. 4). Importantly, resilient chickens exhibited a downregulation of the same genes in both tissues at all analyzed time points compared with controls (Supplementary Fig. 4). Some genes involved in type I interferon response, typically associated with AI infection (e.g., *STAT1, MX1, IFITM5, IFI6*), were significantly upregulated in susceptible birds compared with both control and resilient groups in both tissues and time points (Supplementary Fig. 5). However, no significant differences in the expression of these ISGs were observed between the resilient and control groups. Together, these findings support that susceptible birds exhibit higher viral loads in the respiratory tissues analyzed, accompanied by strong antiviral and inflammatory responses. In contrast, resilient birds display lower viral loads in these tissues and, correspondingly, reduced ISG expression, consistent with a lower magnitude of interferon-mediated signaling.

### HPAIV-resilient chickens exhibit upregulation of adhesion and MAPK signaling pathways, but downregulate antiviral responses

Upon closer examination of the Venn diagram (Fig. 3**B**), we focused on the biological processes of the DEGs identified exclusively in the NT of resilient chickens. Upregulated DEGs in NT involved pathways such as “Focal adhesion” (gga04510), “ECM-receptor interaction” (gga04512), and “MAPK cascade” (GO:0000165) at 48 hpi, and “adherens junction organization” (GO:0034332) at 72 hpi (Supplementary Fig. 6**A**). Downregulated DEGs in the NT at 48 hpi identified terms such as “Influenza A” (gga05164) and “regulation of apoptotic process” (GO:0042981). Moreover, pathways associated with “apoptotic process” (GO:0006915), “positive regulation of toll-like receptor 2 signaling pathway” (GO:0034137), and “regulation of apoptotic process” (GO:0042981) were identified in NT at 72 hpi (Supplementary Fig. 6B). Finally, downregulated DEGs in the lung samples at 48 hpi were associated with the cell cycle and RNA transcription processes (Supplementary Fig. 6B).

**Fig. 4:**
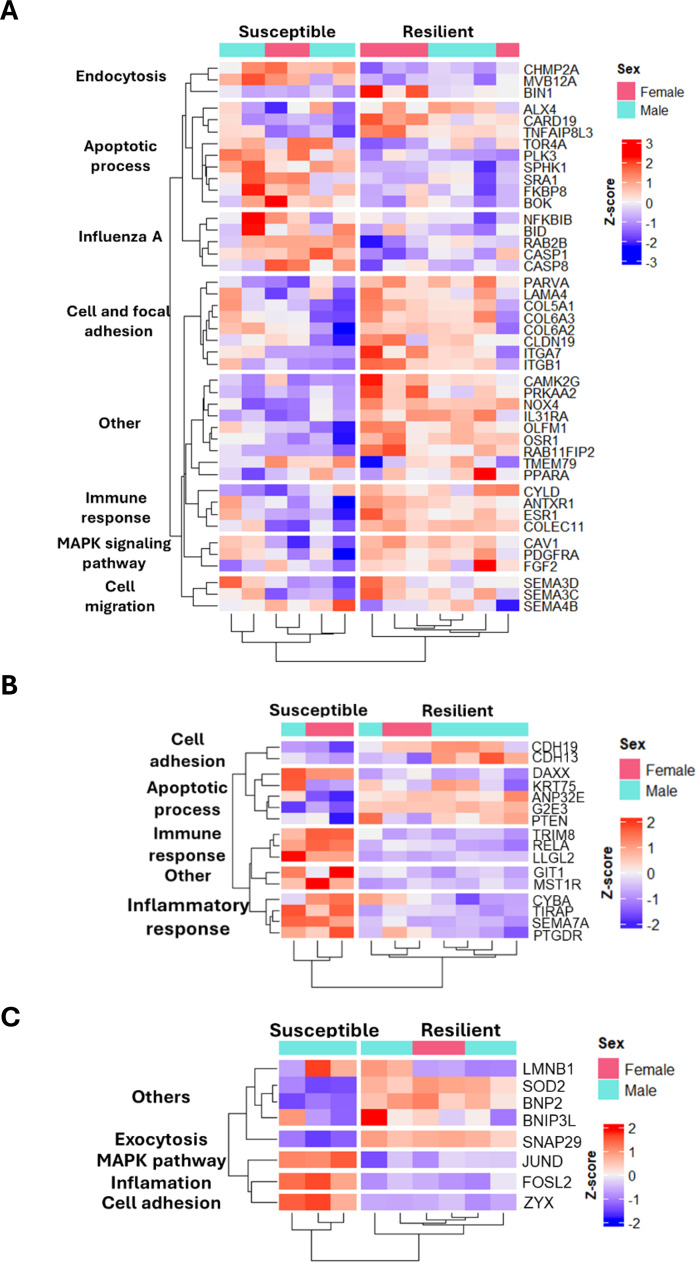
**Transcriptomic signatures of NT (48 and 72 hours post-inoculation (hpi)) and lung (48 hpi) in HPAIV-resilient compared with HPAIV-susceptible chickens. (A)** Heatmap showing normalized RNA-seq expression of DEGs exclusively identified in the NT at 48 hpi, **(B)** in the NT at 72 hpi, and **(C)** in the lung samples of resilient birds at 48 hpi. Rows and columns were hierarchically clustered using the pheatmap package in R.

To illustrate the gene expression patterns underlying the pathways identified in resilient chickens, Fig. 4 shows heatmaps of DEGs in NT and lung tissues. Related to DEGs in NT at 48 hpi, genes associated with MAPK signaling (*CAV1, PDGFRA*, and *FGF2*) and with cell and focal adhesion (*ITGB1, ITGA7, CLDN19, COL6A3*, and *PARVA*) showed higher expression in resilient vs susceptible chickens. In contrast, lower expression levels were observed for genes related to the Influenza A pathway (*NFKBIB, BID, RAB2B, CASP1*, and *CASP8*) and for genes involved in the apoptotic process (*SPHK1, SRA1, FKBP8*, and *BOK*). Nevertheless, within this same category, some genes were upregulated (*ALX4, CARD19*, and *TNFAIP8L3*) (Fig. 4**A**). Differences in the expression patterns of apoptosis-associated genes were also evident in the dendrogram subclusters.

At 72 hpi in the NT, genes associated with the apoptotic process, such as *ANP32E* and *G2E3*, were upregulated in resilient chickens compared with susceptible birds. Conversely, genes involved in immune responses (e.g., *TRIM8* and *RELA*) and inflammatory signaling (e.g., *CYBA, TIRAP, SEMA7A*, and *PTGDR*) were clearly downregulated in resilient birds (Fig. 4**B**).

In lung samples from resilient chickens, several DEGs related to apoptosis, including *SOD2* and *BNIP2*, were also upregulated, together with genes involved in exocytosis (*SNAP29*). In contrast, genes associated with inflammatory responses (*FOSL2*), MAPK signaling (*JUND*), and cell adhesion (*ZYX*) were downregulated compared with susceptible birds (Fig. 4**C**).

**Fig. 5:**
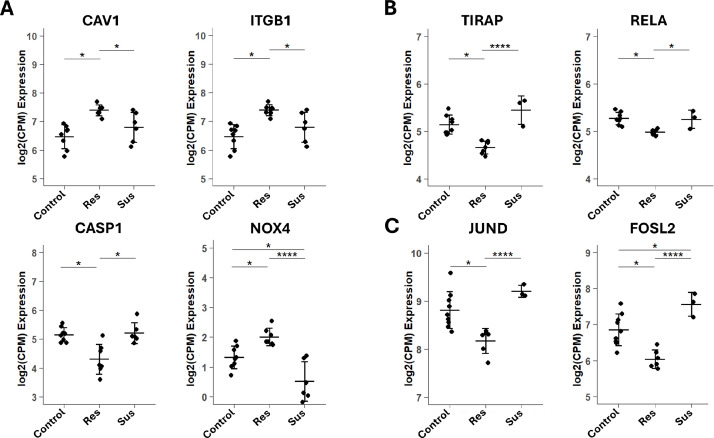
**Expression levels of representative DEGs in HPAIV-resilient chickens. (A)** NT at 48 hours post-inoculation (hpi), **(B)** NT at 72 hpi, and **(C)** lungs at 48 hpi. Plots show RNA-seq–derived expression values (log₂[CPM]) for selected genes. Statistical significance between groups was assessed using limma with empirical Bayes moderation. Adjusted *p*-values: *p* ≤ 0.*0*5 (*), *p* ≤ 0.01 (**), *p* ≤ 0.001 (***), *p* ≤ 0.0001 (****).

Notable DEGs in resilient birds included *CAV1, ITGB1*, and *NOX4* (all upregulated) and *CASP1* (downregulated) in the NT at 48 hpi (Fig. 5**A**). At 72 hpi, *TIRAP* and *RELA* were also downregulated in NT (Fig. 5**B**). In addition, in the lungs at 48 hpi, *JUND* and *FOSL2* were downregulated in resilient birds (Fig. 5**C**). Importantly, all of these genes exhibited differential expression not only between resilient and susceptible chickens but also between resilient and control chickens, supporting their association with a favorable clinical outcome following HPAIV infection. Overall, these transcriptomic results in resilient chickens indicate that respiratory tissues coordinate the regulation of epithelial remodeling and signaling pathways, while, at the same time, limiting excessive innate immune and inflammatory activation, particularly in the NT. This is consistent with a model of more localized and regulated host response at the primary site of infection.

Moreover, expression levels of *CAV1, NOX4*, and *ITGB1* were positively correlated with Ct values in the Spearman correlation analysis, suggesting that higher expression of these genes was associated with lower viral RNA abundance. In contrast, *JUND* showed a negative, although non-significant, correlation with Ct values. These results provide quantitative support for the association between the resilience-associated transcriptional profile and reduced viral burden at the primary site of infection (Supplementary Fig. 7).

### Early immune- and apoptosis-associated signatures in NT and lungs (including MVB12A and RELA) differentiate HPAIV-resilient from -susceptible and control chickens

**Fig. 6:**
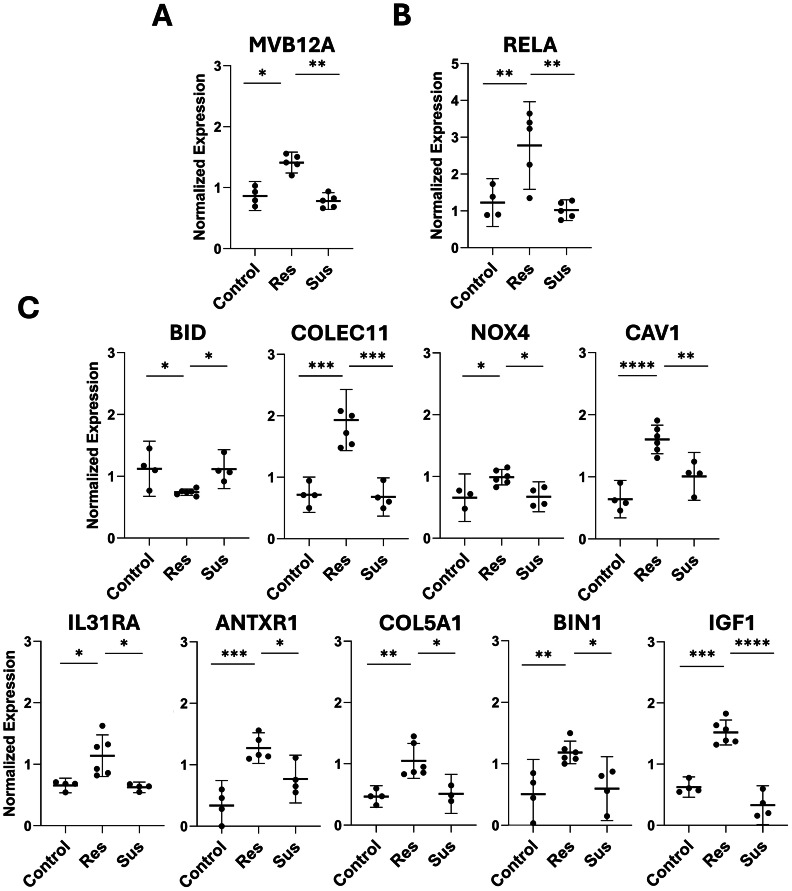
**Gene expression analysis by microfluidic quantitative PCR.** Differentially expressed genes identified in NT at 24 hours post-inoculation (hpi) are shown in panel A, while genes identified in lung at 24 hpi are shown in panel B. Panel C shows the validation of selected genes in NT at 48 hpi using microfluidic quantitative PCR. Values are shown as mean ± SD. Group comparisons were performed using Welch’s ANOVA, and p-values were adjusted for multiple testing using the Benjamini–Hochberg method. Adjusted *p*-values indicating significant differences between groups are represented as follows: *p ≤ 0.05, **p ≤ 0.01, ***p ≤ 0.001, and ****p ≤ 0.0001.

**Fig. 7:**
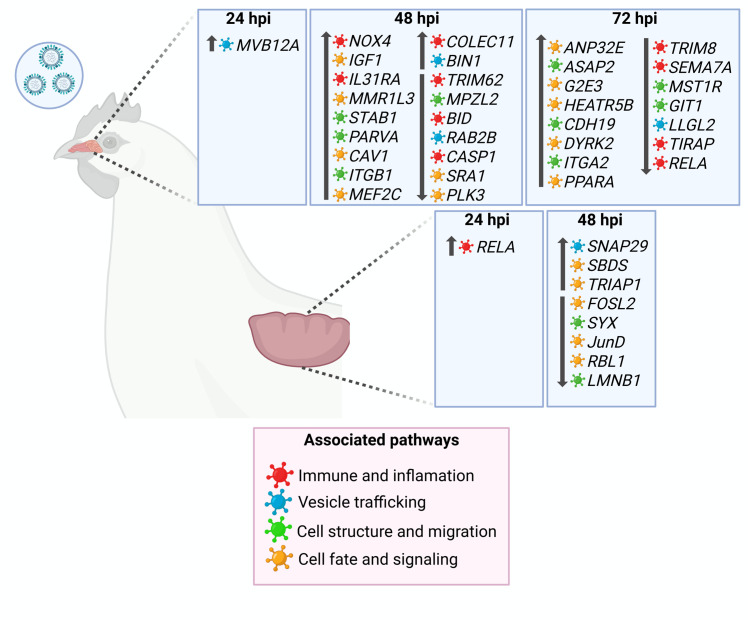
**Schematic representation of DEGs in HPAIV-resilient chickens at 24, 48, and 72 hours post-inoculation (hpi) compared with control and HPAIV-susceptible groups.** The Fig. illustrates a proposed model based on our results in which these genes collectively contribute to the resilience phenotype. Genes are shown by tissue and time point and are classified into major functional categories, including immune and inflammatory responses, vesicle trafficking, cell structure and migration, and cell fate and signaling, as indicated by color coding. Arrows denote the direction of differential expression (up or down regulation) in HPAIV-resilient chickens relative to the control group. Created with BioRender.com.

A total of 44 genes were analyzed by microfluidic quantitative PCR to validate the transcriptomic findings and to further characterize gene expression in tissues prior to the onset of clinical signs (Supplementary Table 2). Gene expression was evaluated in NT and lung samples collected at 24 hpi, and in NT samples collected at 48 hpi (Supplementary Fig. 8). At 24 hpi, resilient chickens were distinguished from both control and susceptible birds by differential expression of two genes (*MVB12A* in NT; *RELA* in lungs) (Fig. 6**A, B**). Additionally, DEGs identified at 48 hpi with significant differences in resilience compared with susceptible and controls were also validated by microfluidic quantitative PCR (Fig. 6**C**). Together, these results suggest that divergent clinical outcomes are associated with detectable transcriptional differences in the upper respiratory tract as early as 24 hpi, specifically in immune and apoptosis genes. A summary of the main genes identified across tissues and time points is presented in Fig. 7.

## Discussion

Our findings indicate that resilience to HPAIV infection in chickens is unlikely to depend on the action of single genes. Instead, it appears to arise from the coordinated regulation of multiple host-response pathways, supporting the idea that the host response to infection is a complex and multifactorial process with a strong genetic component (Drobik-Czwarno et al., 2018, 2024). In this respect, some studies have found genes associated with resilience to HPAIV infection in chickens (Bryson et al., 2023; Perlas et al., 2021; Vu et al., 2022, 2023), but none have thoroughly investigated tissues of the primary site of replication, upper respiratory tract, along with the lung, at early time points. This study investigates transcriptomic changes in the NT and lungs in resilient and susceptible broiler chickens at early time points post-H7N1 HPAIV inoculation. We identify genes and pathways that may explain why some chickens survive HPAIV infection while others die.

Although viral RNA levels were low in resilient birds, the fact that their transcriptomic profiles differed from those of controls is consistent with viral exposure and a host response associated with a favorable clinical outcome, rather than intrinsic resistance or absence of infection. This observation is important when distinguishing resilience from resistance in the context of HPAIV infection. Resistance refers to the ability to prevent infection or fully block viral replication, whereas resilience describes the capacity of the host to remain clinically stable despite infection by limiting viral burden and tissue damage (Chen et al., 2025). Our data indicate that the presence of viral RNA in tissues, detected by RNA-seq and RT-qPCR, is consistent with exposure to infection in resilient birds. Moreover, both viral RNA levels and viremia tended to decrease between 48 and 72 hpi in resilient chickens, which is consistent with a temporal reduction in viral presence.

Our findings are consistent with a model in which clinical outcome is associated with the spatial and temporal dynamics of host response. It is worth noting that, since birds were classified based on later clinical outcome, the transcriptional differences identified at 48 and 72 hpi should be interpreted primarily as outcome-associated signatures rather than as definitive drivers of resilience or susceptibility. Birds that were susceptible showed extensive transcriptional activation in both NT and lungs, consistent with the virus spreading rapidly beyond the upper respiratory tract (Burggraaf et al., 2014; Kalaiyarasu et al., 2016). In contrast, resilient chickens exhibited more localized and regulated responses at the primary site of infection, with transcriptional changes more evident in the nasal mucosa within the analyzed time points.

The predominance of transcriptomic changes within the analyzed datasets suggests that the nasal mucosa may play an important role in early defense against HPAIV infection, where the nasal-associated lymphoid tissue both recognizes the antigen and initiates the immune response (Kang et al., 2013; Śmiałek et al., 2011; Wang et al., 2020). Despite the limited number of DEGs identified in resilient chickens, differences in clinical outcome were associated with regulatory dynamics of host-response pathways. Accordingly, the transcriptional changes identified in the nasal mucosa were more focal and consistent with a more contained infection, as reflected by pathway-associated gene expression patterns related to the MAPK cascade, apoptosis, inflammatory response, and antiviral pathways. However, it is worth highlighting that the absence of lung samples at 72 hpi limits comparisons between tissues at later stages of infection.

Functional enrichment analysis confirmed that both resilient and susceptible chickens activate an immune response following HPAIV infection. However, susceptible birds exhibited a more intense innate immune response in both NT and samples at 48 and 72 hpi, whereas resilient birds showed a more regulated transcriptional profile, with expression levels of several ISGs (e.g., *IFIT5, IFITM5, IFITM10, STAT2*) in the NT at 48 hpi that tended to be lower than in controls and were clearly reduced compared with susceptible birds, suggesting a more limited inflammatory activation in resilient birds. This pattern is consistent with the lower viral loads in the upper respiratory tract of these birds and suggests that the magnitude of ISG activation may largely reflect differences in viral burdens rather than intrinsic differences in antiviral signaling capacity. Importantly, the same H7N1 HPAIV strain and intranasal inoculation technique have been used repeatedly by our group and consistently produce a mixed clinical outcome under identical conditions, supporting the robustness of the experimental infection model (Bertran et al., 2013; Perlas et al., 2021; Sánchez-González et al., 2020).

Focusing on the pathways in HPAIV-resilient chickens, our results suggest that resilience may depend more on a coordinated regulation of inflammatory signaling, epithelial integrity, and apoptosis than on the activity of individual genes, consistent with previous reports indicating that resistance to HPAIV involves multiple genes (Drobik-Czwarno et al., 2018, 2024). One pathway of interest identified in resilient chickens was the MAPK signaling, which mediates the inflammatory response during HPAIV infection (Tisoncik et al., 2012; Yu et al., 2020). Furthermore, the differential expression of *CAV1* and *NOX4* in the NT and *JUND* in the lung at 48 hpi may reflect tissue-specific modulation of MAPK-related signaling during the host response to infection (Bohm et al., 2014; Chen et al., 2012; Hendricks et al., 2022; Sun et al., 2010).

Focal and cell adhesion pathways, represented by *ITGB1, PARVA, LAMA4*, and *COL5A1*, were also identified in resilient chickens at 48 hpi. These genes are associated with pathways involved in tissue integrity and cellular stability during infection and, therefore, may indicate a more robust tissue condition in resilient birds (Aman and Margadant, 2023). However, since the data were generated from bulk RNA-seq, the specific cellular origin of these transcripts cannot be determined. Moreover, several genes associated with the Influenza A pathway and apoptotic signaling, including *BID, CASP1*, and *CASP8*, were downregulated in the NT at 48 hpi. Apoptosis, commonly activated during early stages of influenza infection, contributes to both antiviral defense and tissue damage (Othumpangat et al., 2018). Thus, reduced expression of pro-apoptotic mediators such as *BID* may therefore reflect a more controlled regulation of cell death pathways in resilient birds, consistent with their lower viral burden and the corresponding reduced induction of ISGs (Wyżewski et al., 2025; Zamarin et al., 2005). It is worth highlighting that genes like *CAV1* and *BID* depend on post-translational processes (e.g., proteolytic cleavage and subcellular localization); therefore, transcriptomic data alone cannot fully resolve the functional roles of these genes, and additional functional studies are required to clarify their contributions during HPAIV infection (Parat and Fox, 2001; Zha et al., 2000). For instance, siRNA-mediated knockdown of *CAV1* could help determine its role in viral entry and replication, while approaches such as single-cell RNA sequencing (scRNA-seq) may further resolve cell-type–specific responses (Dai et al., 2023).

Understanding the transcriptomic changes in resilient and susceptible chickens before any clinical manifestation could help to identify the earliest differential responses associated with divergent clinical outcomes in HPAIV-infected chickens. In this context, DEGs in NT (*MVB12A*) and lung (*RELA*) samples were detected at 24 hpi, before the onset of clinical signs. *MVB12A* is involved in the ESCRT machinery, which participates in the budding of enveloped viruses (Calistri et al., 2021), whereas *RELA* is a component of the NF-κB signaling pathway and participates in antiviral and inflammatory responses (Basagoudanavar et al., 2011; Kumar et al., 2008). The limited number of DEGs identified at 24 hpi between groups likely reflects the early stage of infection. A whole-transcriptomic analysis at this early time point would likely provide a more comprehensive picture of early molecular differences associated with later clinical outcomes. Although these genes represent potential markers of disease outcome following HPAIV infection in chickens, functional studies are required to confirm a causal relationship.

Moreover, a major limitation of this study is the small sample size for susceptible birds at later time points. This limitation likely reduced the statistical power to detect DEGs and significantly enriched pathways. For this reason, the interpretation of genes or pathways appearing as uniquely associated with resilience should be approached with caution. This concern is particularly relevant for lung analysis at 48 hpi and NT at 72 hpi, where the reduced number of susceptible samples may have influenced the robustness of the results. This limitation also affects the interpretation of the PCA; thus, results should be interpreted in the context of the modest sample size. A further limitation is that all birds received an infectious bronchitis virus vaccine at 1 day of age. Although this was consistent across all experimental groups, prior vaccination may have influenced the birds' immune status and potentially modulated their systemic and local transcriptional responses to HPAIV infection. Therefore, the potential confounding effect of this vaccination should be considered when interpreting these results. Another limitation is that uninfected controls were only sampled at baseline (0 hpi). However, comparisons between resilient and susceptible birds were performed at matched time points, minimizing the influence of potential age-related transcriptional changes.

Overall, this study aimed to deepen our understanding of host resilience mechanisms against HPAIV infection in chickens by characterizing the early molecular response occurring in the upper respiratory tract. While we identified promising pathways (apoptotic process, cell adhesion, MAPK signaling, and inflammatory response) and genes (*CAV1, ITGB1*, and *NOX4*) as candidates for further investigation, others (e.g., *MVB12A, ANP32E, G2E3, TRIM8*) should be interpreted more cautiously and will require additional studies. Functional validation using CRISPR/Cas9 gene editing or siRNA-mediated knockdown approaches will be essential to determine the functional roles of these candidates. In conclusion, our results identify early immune and cellular responses in the NT and lungs associated with resilience to HPAIV infection and provide a foundational dataset for exploring breeding or gene-editing strategies to enhance genetic resilience in poultry.

## Data availability statement

The raw transcriptomic data are available in NCBI SRA under the BioProject accession number PRJNA1413929.

## Ethics statement

The experimental design was approved by the Ethics Committees on Animal Experimentation of the institution and the Catalan Government (CEA-OH/11722/2).

## Author Contributions

The project was conceived, and funding was obtained by NM and KB. The experiment was designed by NM and KB. The experiment was performed by MJV-M, NM, and KB, and samples were collected and processed by MJV-M, MN, RV, and MP. MJV-M, MD, AE-C, and JA assisted with the RNA-seq analysis. MJV-M, SP-P, and MP performed microfluidic quantitative PCR. MJV-M, JA, and KB prepared the figures. MJV-M drafted the manuscript with corrections from NM and KB. All authors reviewed and approved the manuscript. The authors declare no competing interests.

## Funding

This work has been funded by the 10.13039/501100004837Spanish Ministry of Science and Innovation and was supported by the coordinated project PID2020-114060RR-C33-INFLUOMA, entitled “Unravelling the molecular mechanisms of avian influenza virus infection outcome in the avian host by using a multi-omics approach”. MJV-M is a recipient of a Secretariat of Science, Humanities, Technology and Innovation of Mexico (SECIHTI, CVU 1070914). KB is funded by the Ministry of Economy and Competitiveness, Spain, Programa Ramón y Cajal (Grant RYC2021-033472-I).

## Acknowledgments

We gratefully acknowledge Dr. Maria Ballester for their assistance in designing the PCR Multiplex Fluidigm. We also thank the BSL-3 staff at IRTA-CReSA for all their support and help during the experiments.

Institutional support to CNAG was provided by the Spanish Ministry of Science and Innovation through the Instituto de Salud Carlos III, and by the Generalitat de Catalunya through the Departament de Salut and the Departament de Recerca i Universitats.

## Disclosures

The authors declare that they have no known competing financial interests or personal relationships that could have appeared to influence the work reported in this paper.

## Appendix Supplementary materials

Image, application 1

Image, application 2

Image, application 3

Image, application 4

Image, application 5

Image, application 6

Image, application 7

Image, application 8

Image, application 9

Image, application 10

## Footnotes

Supplementary material associated with this article can be found, in the online version, at doi:10.1016/j.psj.2026.106833.
