# Supplementary material for: Early nasal and lung transcriptomic profiles reveal pathways associated with divergent clinical outcomes following H7N1 high pathogenicity avian influenza virus infection
Source: Poult Sci. 2026 Mar 20;105(7):106833. doi: 10.1016/j.psj.2026.106833 (PMC13098617; doi:10.1016/j.psj.2026.106833)
Supplement: Supplementary file 3 [file mmc3.docx]

**
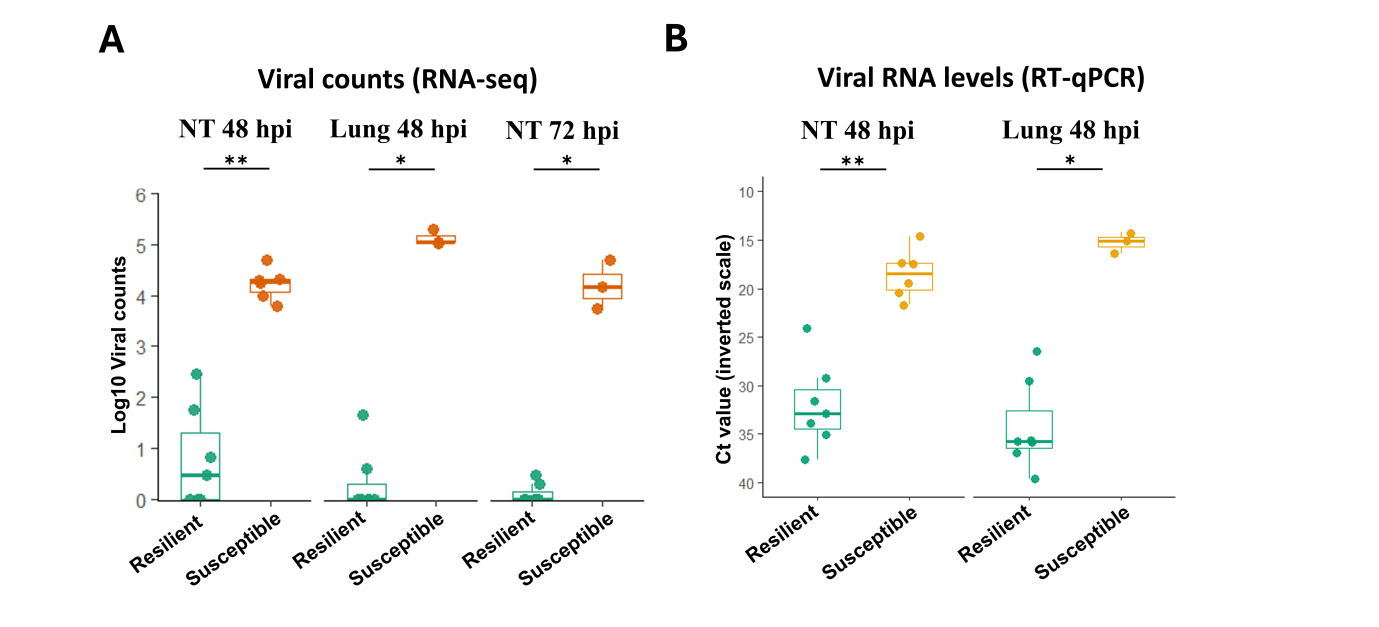
**

**Supplementary Figure 1**. **Viral RNA detection in tissues of HPAIV-susceptible and HPAIV-resilient chickens. (A)** Viral RNA counts obtained from RNA-seq in NT at 48 and 72 hours post-inoculation (hpi), and lungs at 48 hpi. The viral read counts are shown as log_10_-transformed. **(B)** Viral RNA levels measured by RT-qPCR in NT and lungs at 48 hpi, expressed as CT values. Each dot represents an individual bird, and boxes indicate the interquartile range with the median. Statistical differences between groups were assessed using the Wilcoxon rank-sum test. P-values: p ≤ 0.*0*5 (*), p ≤ 0.01 (****),** p **≤ 0.001 (*****), p ≤ 0.0001 (****).
