# Supplementary material for: Early nasal and lung transcriptomic profiles reveal pathways associated with divergent clinical outcomes following H7N1 high pathogenicity avian influenza virus infection
Source: Poult Sci. 2026 Mar 20;105(7):106833. doi: 10.1016/j.psj.2026.106833 (PMC13098617; doi:10.1016/j.psj.2026.106833)
Supplement: Supplementary file 4 [file mmc4.docx]

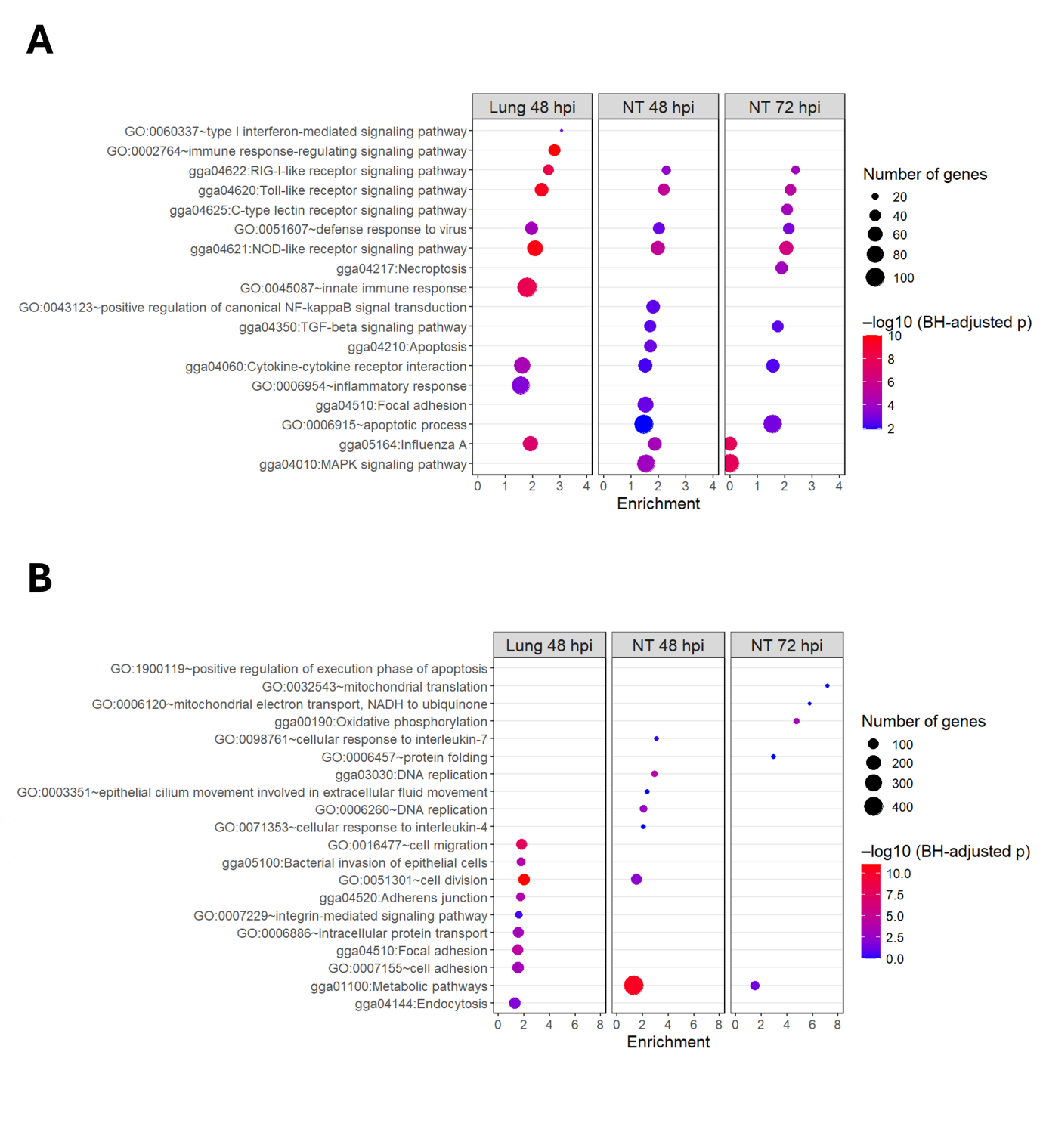


**Supplementary Figure** **2**. **Functional enrichment analysis of upregulated (A) and downregulated (B) differentially expressed genes (DEGs) in NT and lungs of HPAIV-susceptible chickens collected at 48 and 72 hours post-inoculation (hpi).** The dot color indicates the –log10 of the Benjamini–Hochberg adjusted *p*-value, and the dot size corresponds to the number of DEGs associated with each GO term. Selected GO terms relevant to the biological processes under study are shown, although not all terms remained statistically significant after multiple testing correction.
