# Supplementary material for: Early nasal and lung transcriptomic profiles reveal pathways associated with divergent clinical outcomes following H7N1 high pathogenicity avian influenza virus infection
Source: Poult Sci. 2026 Mar 20;105(7):106833. doi: 10.1016/j.psj.2026.106833 (PMC13098617; doi:10.1016/j.psj.2026.106833)
Supplement: Supplementary file 5 [file mmc5.docx]

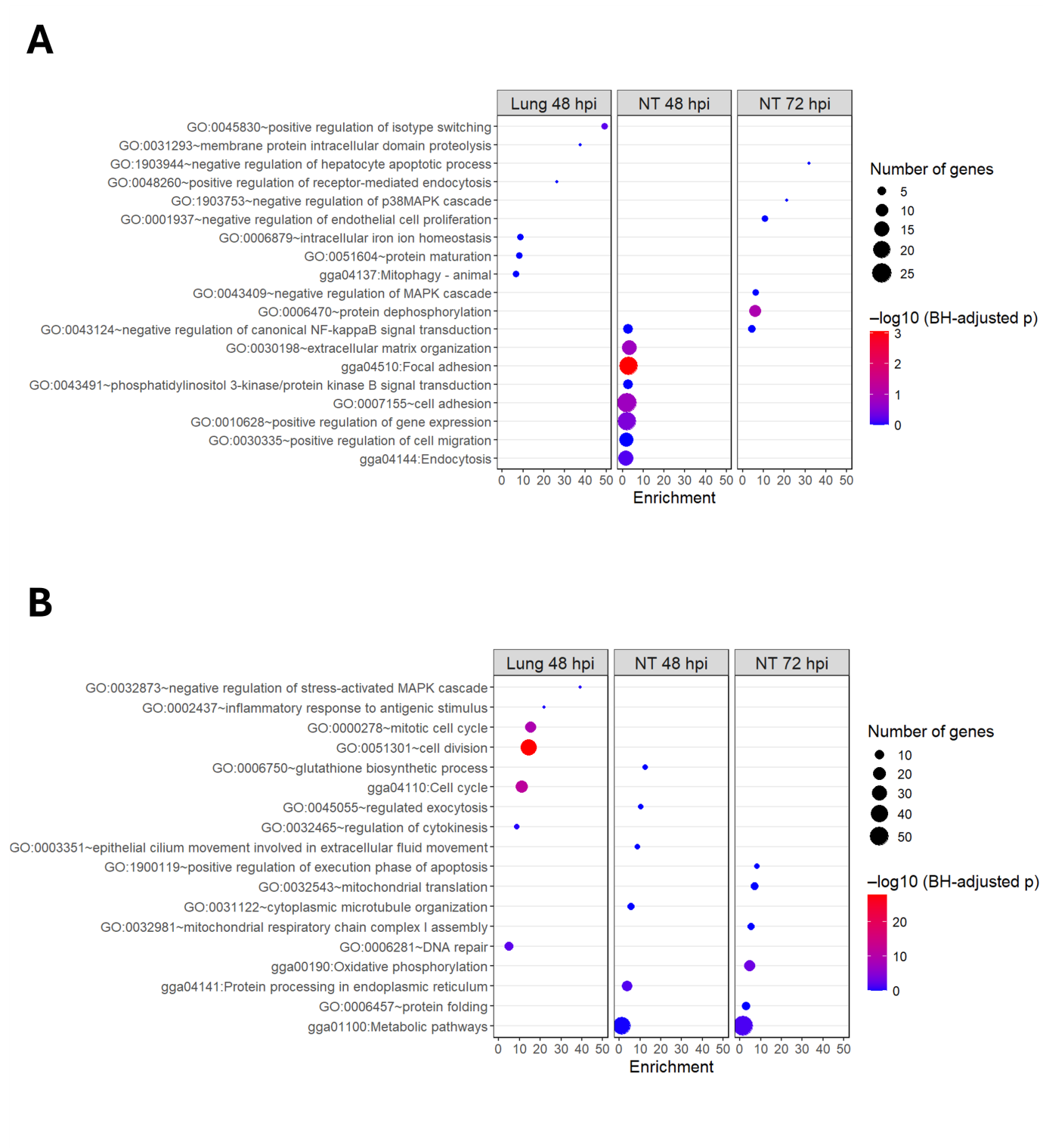


**Supplementary Figure 3**. **Functional enrichment analysis of upregulated (A) and downregulated (B) differentially expressed genes (DEGs) in NT and lungs of HPAIV-resilient chickens collected at 48 and 72 hours post-inoculation (hpi).** Dot color represents –log10 of the Benjamini–Hochberg-adjusted p-value, and dot size corresponds to the number of DEGs associated with each term or pathway. Selected terms and pathways relevant to the biological processes under study are shown, although not all terms remained statistically significant after multiple testing correction.
