# Supplementary material for: Early nasal and lung transcriptomic profiles reveal pathways associated with divergent clinical outcomes following H7N1 high pathogenicity avian influenza virus infection
Source: Poult Sci. 2026 Mar 20;105(7):106833. doi: 10.1016/j.psj.2026.106833 (PMC13098617; doi:10.1016/j.psj.2026.106833)
Supplement: Supplementary file 6 [file mmc6.docx]

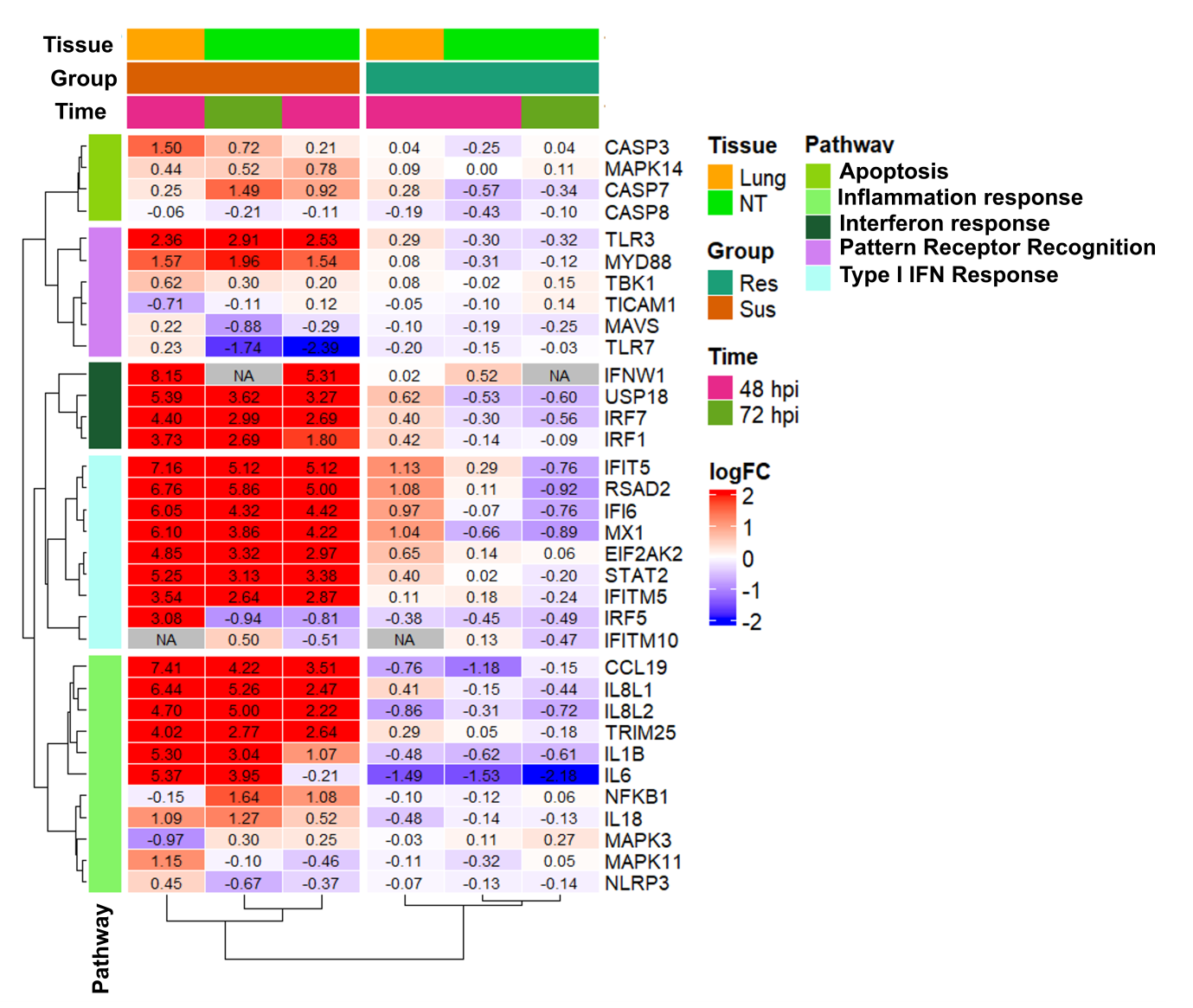


**Supplementary Figure 4. Nasal turbinate (NT) and lung transcriptomic signatures at 48 and 72 hours post-inoculation (hpi) in HPAIV-resilient and HPAIV-susceptible chickens.** The heatmap shows the log2 fold change (log2FC) values of RNA-seq expression levels of selected differentially expressed genes (DEGs) in HPAIV-resilient and HPAIV-susceptible groups compared to controls. The heatmap was created using the R package *pheatmap*.
