# Supplementary material for: Early nasal and lung transcriptomic profiles reveal pathways associated with divergent clinical outcomes following H7N1 high pathogenicity avian influenza virus infection
Source: Poult Sci. 2026 Mar 20;105(7):106833. doi: 10.1016/j.psj.2026.106833 (PMC13098617; doi:10.1016/j.psj.2026.106833)
Supplement: Supplementary file 7 [file mmc7.docx]

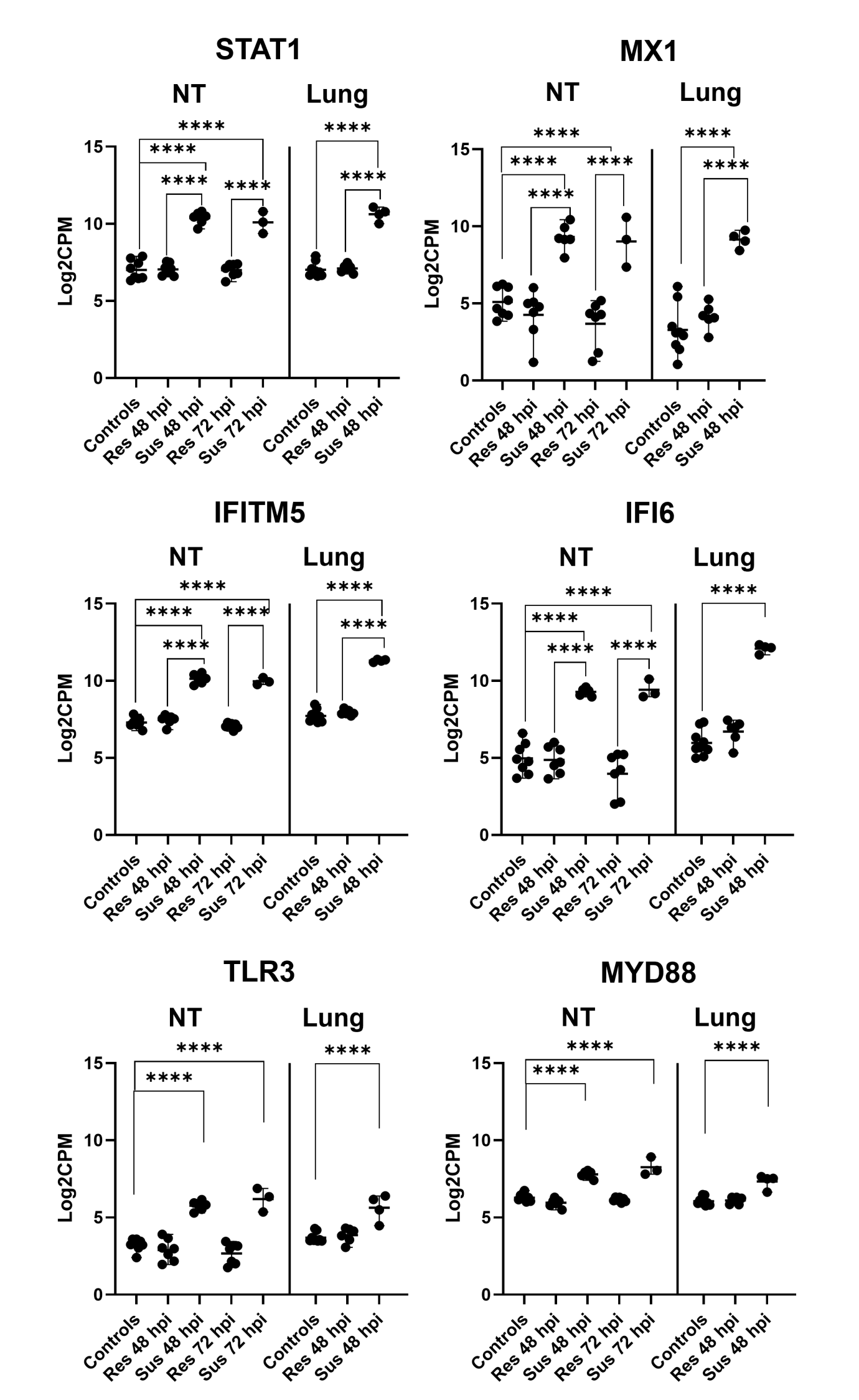


**Supplementary Figure 5. Gene expression levels of IFN-I and inflammation-related genes in NT and lung samples collected at 48 and 72 hours post-inoculation (hpi) from control,** **HPAIV-resilient, and HPAIV-susceptible chickens.** RNA-seq-derived expression levels of selected genes in each category are presented as log2 CPM values, obtained using the limma-voom method. Data are presented as mean, and standard deviation. Statistical significance between groups was assessed using limma with empirical Bayes moderation, and *p*-values were adjusted for multiple testing. Significance is indicated as follows: **p* ≤ 0.05, ***p* ≤ 0.01, ****p* ≤ 0.001, and *****p* ≤ 0.0001.
