# Supplementary material for: Early nasal and lung transcriptomic profiles reveal pathways associated with divergent clinical outcomes following H7N1 high pathogenicity avian influenza virus infection
Source: Poult Sci. 2026 Mar 20;105(7):106833. doi: 10.1016/j.psj.2026.106833 (PMC13098617; doi:10.1016/j.psj.2026.106833)
Supplement: Supplementary file 8 [file mmc8.docx]

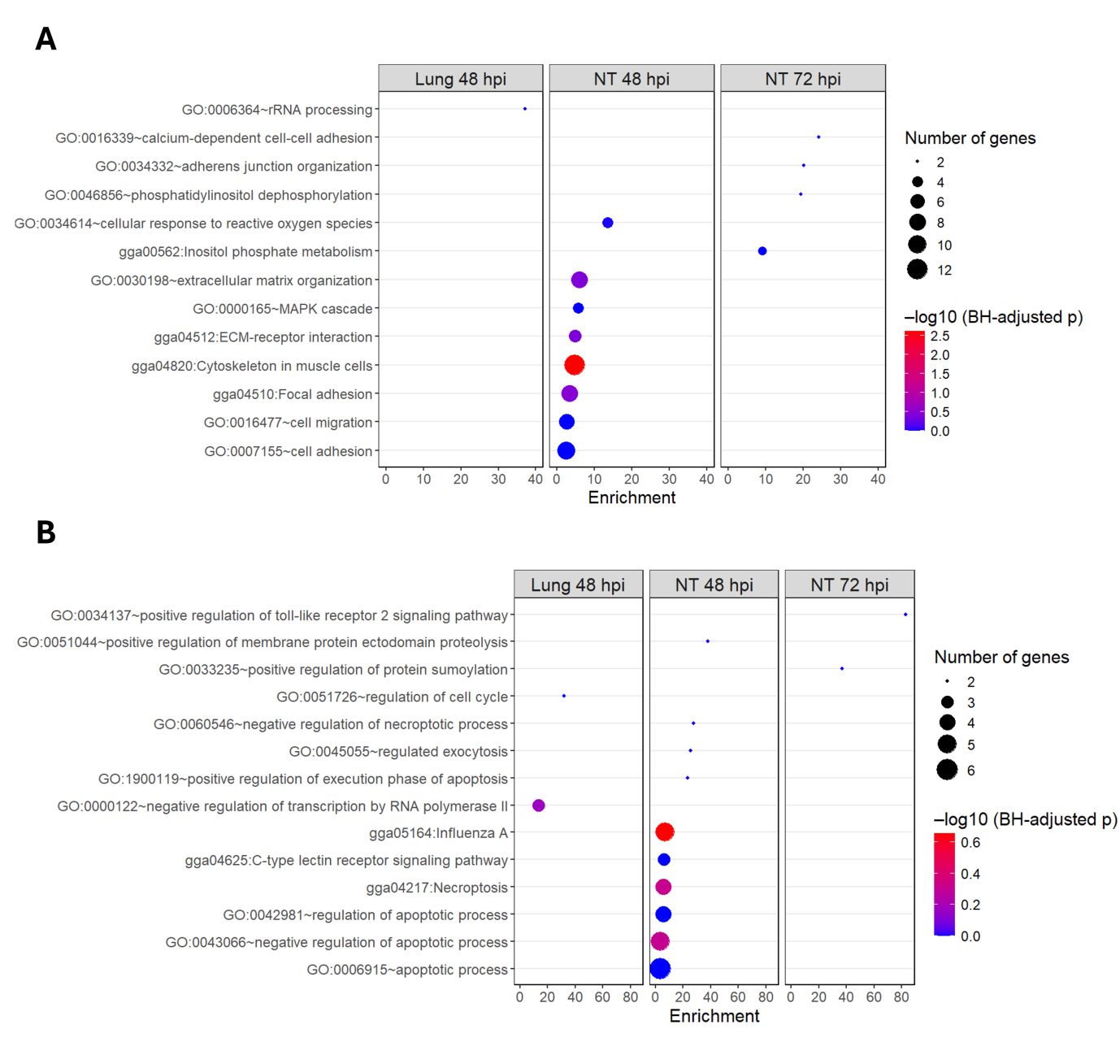


**Supplementary Figure 6. Functional enrichment analysis of upregulated (A) and downregulated (B) differentially expressed genes (DEGs) identified exclusively in HPAIV-resilient chickens.** This analysis was performed using DEGs from NT at 48 and 72 hours post-inoculation (hpi) (NT 48 hpi and NT 72 hpi), and from lungs at 48 hpi (lung 48 hpi). Dot color represents –log10 of the Benjamini–Hochberg-adjusted p-value, and dot size corresponds to the number of DEGs associated with each term or pathway.
