# Supplementary material for: Early nasal and lung transcriptomic profiles reveal pathways associated with divergent clinical outcomes following H7N1 high pathogenicity avian influenza virus infection
Source: Poult Sci. 2026 Mar 20;105(7):106833. doi: 10.1016/j.psj.2026.106833 (PMC13098617; doi:10.1016/j.psj.2026.106833)
Supplement: Supplementary file 9 [file mmc9.docx]

**
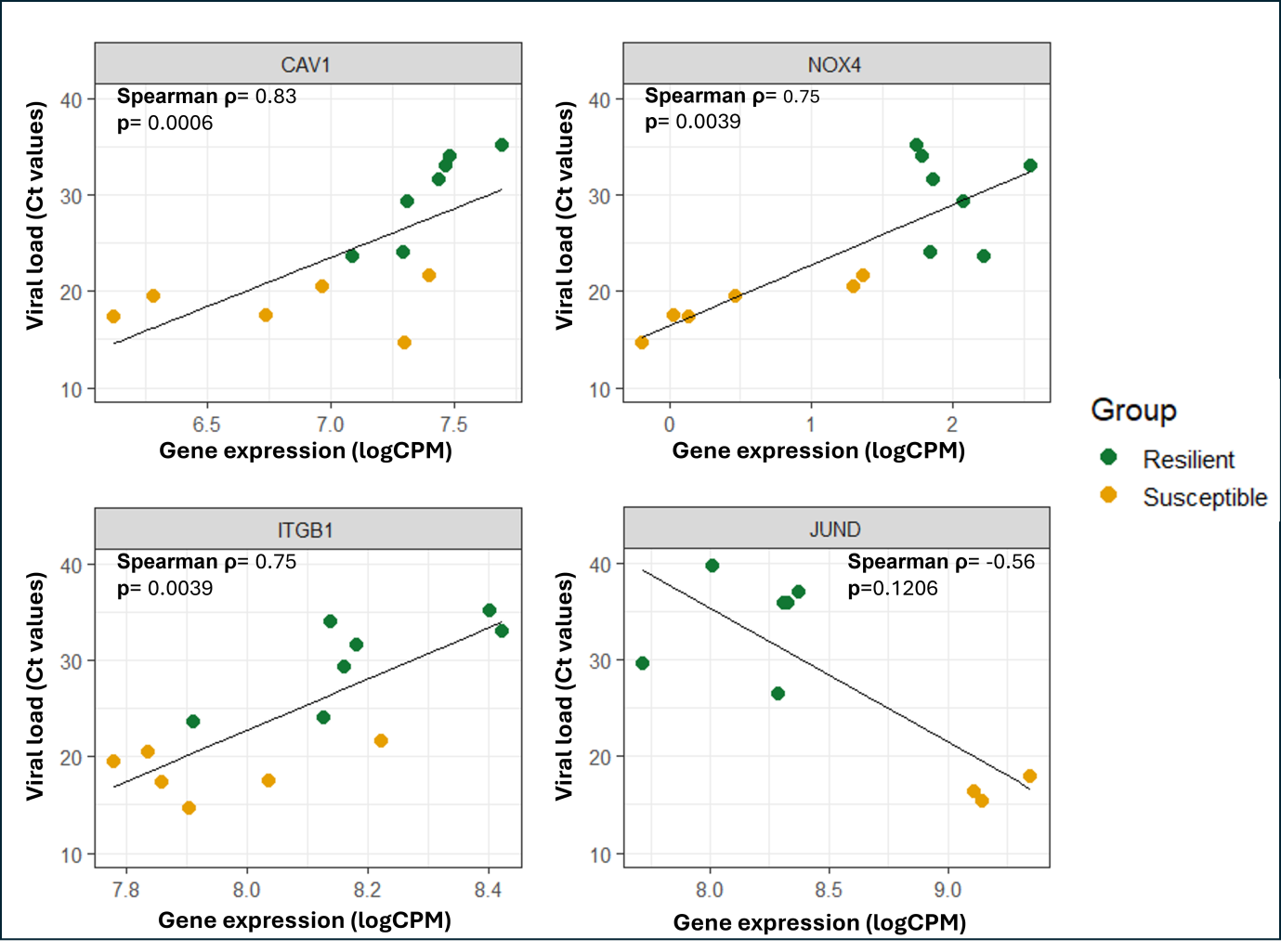
**

**Supplementary Figure 7. Correlation between selected genes and viral RNA levels in NT and lung samples at 48 hours post-inoculation (hpi).** Scatter plots show the relationship between gene expression (*CAV1*, *NOX4*, and *ITGB1* in NT; *JUND* in lung), expressed as log2 CPM, and viral RNA levels estimated from RT-qPCR Ct values in the corresponding tissue at 48 hpi. Each point represents one sample and is colored according to clinical outcome group (resilient in green, susceptible in yellow). Spearman’s rank correlation coefficient (ρ) and corresponding *p*-value are indicated in each panel. Because Ct values are inversely related to viral RNA abundance, positive correlations indicate that higher gene expression is associated with lower viral RNA levels. The line is included to illustrate the overall trend.
