# Supplementary material for: Early nasal and lung transcriptomic profiles reveal pathways associated with divergent clinical outcomes following H7N1 high pathogenicity avian influenza virus infection
Source: Poult Sci. 2026 Mar 20;105(7):106833. doi: 10.1016/j.psj.2026.106833 (PMC13098617; doi:10.1016/j.psj.2026.106833)
Supplement: Supplementary file 10 [file mmc10.docx]

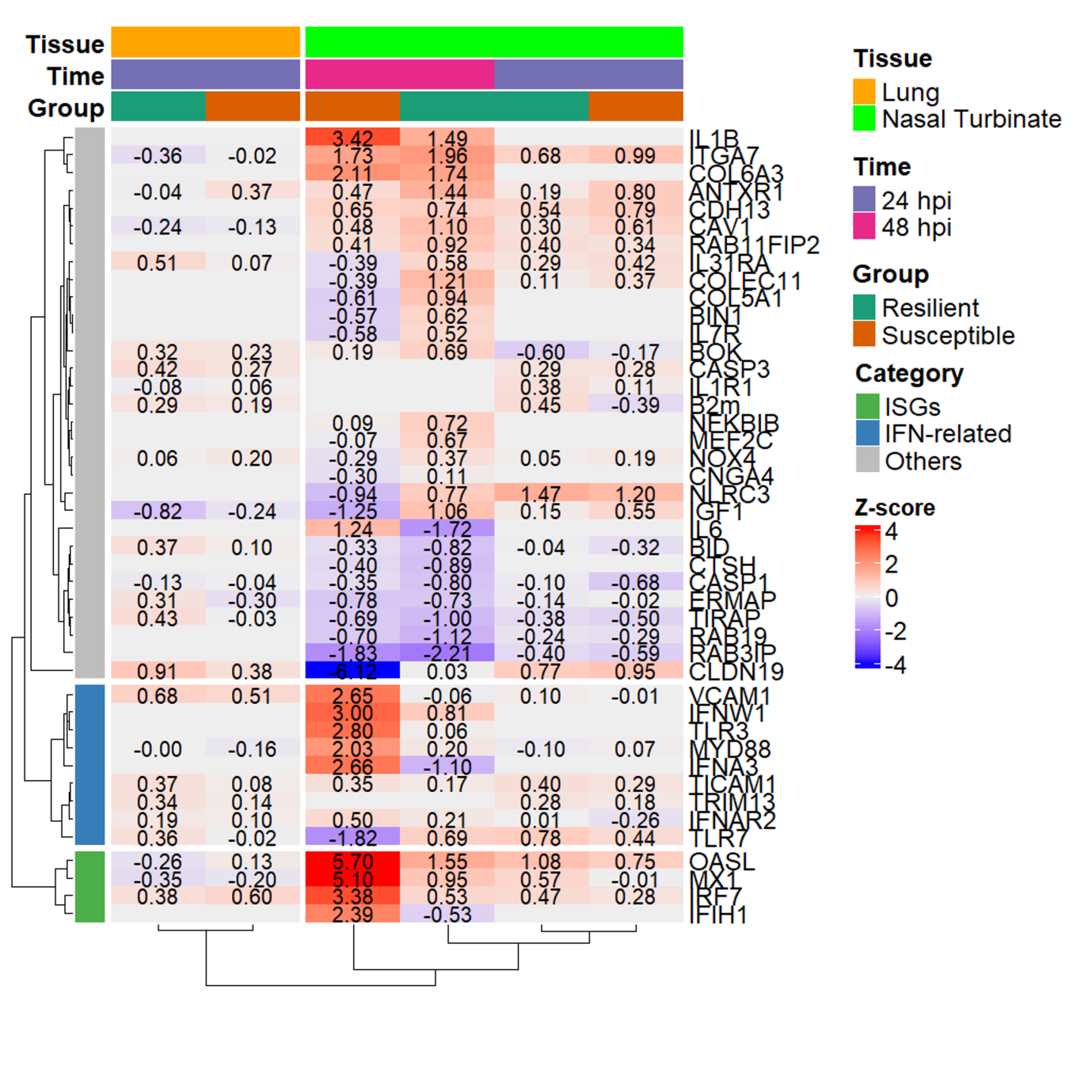
**Supplementary Figure 8. Gene expression analysis by microfluidic quantitative PCR in HPAIV-resilient (Res) and HPAIV-susceptible (Sus) chickens in NT and lung samples at 24 and 48 hours post-inoculation (hpi) compared to controls.** The heatmap displays normalized log2 fold change (log2FC) gene expression values across groups compared to controls. Red indicates upregulation and blue indicates downregulation. Gray boxes represent genes that were not included in the group. Both genes (rows) and sample groups (columns) were clustered using hierarchical clustering.
